# Risk factors for exclusive breastfeeding lasting less than two months—Identifying women in need of targeted breastfeeding support

**DOI:** 10.1371/journal.pone.0179402

**Published:** 2017-06-14

**Authors:** Karin Cato, Sara M. Sylvén, Johan Lindbäck, Alkistis Skalkidou, Christine Rubertsson

**Affiliations:** 1 Department of Women´s and Children´s Health, Uppsala University, Uppsala, Sweden; 2 Department of Neuroscience, Psychiatry, Uppsala University, Uppsala, Sweden; 3 Uppsala Clinical Research Center, Uppsala University, Uppsala, Sweden; Boston University, UNITED STATES

## Abstract

**Background:**

Breastfeeding rates in Sweden are declining, and it is important to identify women at risk for early cessation of exclusive breastfeeding.

**Objective:**

The aim of this study was to investigate factors associated with exclusive breastfeeding lasting less than two months postpartum.

**Methods:**

A population-based longitudinal study was conducted at Uppsala University Hospital, Sweden. Six hundred and seventy-nine women were included in this sub-study. Questionnaires were sent at five days, six weeks and six months postpartum, including questions on breastfeeding initiation and duration as well as several other background variables. The main outcome measure was exclusive breastfeeding lasting less than two months postpartum. Multivariable logistic regression analysis was used in order to calculate adjusted Odds Ratios (AOR) and 95% Confidence Intervals (95% CI).

**Results:**

Seventy-seven percent of the women reported exclusive breastfeeding at two months postpartum. The following variables in the multivariate regression analysis were independently associated with exclusive breastfeeding lasting less than two months postpartum: being a first time mother (AOR 2.15, 95% CI 1.32–3.49), reporting emotional distress during pregnancy (AOR 2.21, 95% CI 1.35–3.62) and giving birth by cesarean section (AOR 2.63, 95% CI 1.34–5.17).

**Conclusions:**

Factors associated with shorter exclusive breastfeeding duration were determined. Identification of women experiencing emotional distress during pregnancy, as well as scrutiny of caregiving routines on cesarean section need to be addressed, in order to give individual targeted breastfeeding support and promote longer breastfeeding duration.

## Introduction

It is well-known that breastfeeding benefits both mothers and infants [1]. Breastfeeding recommendations issued by the World Health Organization (WHO) are to breastfeed exclusively during the first six months and continue breastfeeding with additional food up to two years or beyond. Exclusive breastfeeding (EBF) is defined as giving the infant only human milk, without any additional food or drink, not even water with the exception of oral rehydration solutions, or drops/syrups of vitamins, minerals or medicines [2]. Swedish mothers are recommended to breastfeed exclusively during the first six months, but from the age of four months, an infant interested in solid foods can be offered tiny little tastes as long as they don’t compete with breastfeeding [3]. Healthcare professionals in facilities providing maternity services and care for infants should give mothers information about breastfeeding and support the initiation of breastfeeding within half an hour of birth [4], as timing of the first breastfeeding session after birth seems to have an impact on breastfeeding duration [5–7]. Breastfeeding and breastfeeding duration, in particular, are negatively associated with socio-demographic variables [8–10], depression during pregnancy [11–13], and obstetric variables such as the use of epidural anesthesia [14] and way of giving birth [11].

Breastfeeding rates in Sweden are declining [15]. In the year of 2000 approximately 80% of all infants were exclusively breastfed at the age of two months. This decreased to below 70% in the year of 2012 [15]. It is important to identify factors contributing to shorter EBF duration, in order to provide evidence-based support to breastfeeding mothers. Among those, and apart from maternal characteristics, factors related to the organization of obstetric care might be of importance. In many obstetrical units in Sweden, as well as in the unit in Uppsala, where the current study was carried out, the delivery and the maternity wards are separated. Cesarean sections are carried out in a separate operation ward, and at the time of this study almost all babies born by cesarean section were separated from their mother for several hours and cared for by the mother’s partner in the maternity ward. After a normal birth, without any complications, where the mother and infant have experienced a successful first breastfeeding, the new families can leave the delivery ward as early as six hours after birth, receiving further postpartum care at home by a midwife making home visits. All other families are recommended to stay in the maternity ward for further observation and care. The first breastfeeding session is almost always carried out under supervision of a midwife or a midwife’s assistant, in order to determine the need for further support. In a recent article by our group, the use of the hands-on approach, when healthcare professionals stimulate latch on and breastfeeding by touching the woman’s breast and the baby, was a rather common practice during the first breastfeeding session and was also associated with a more negative experience of the first breastfeeding session [16]. Long-term consequences of receiving the hands-on approach during the first breastfeeding session has, to our knowledge, not been extensively investigated.

The aim of this study was to investigate factors independently associated with a higher risk of EBF lasting less than two months postpartum, and via the use of a nomogram, we hope to provide a tool helping healthcare professionals predict the probability of EBF for more than two months.

## Material and methods

### Design and setting

The present study was based on analyses of data from the UPPSAT study (Uppsala-Athens Project on Postnatal Depression), a population-based cohort in Uppsala, Sweden. For the current study, we used a descriptive, multivariable design to determine which independent variables who were the best predictors of exclusive breastfeeding lasting less than two months postpartum. The study was conducted at the Uppsala University Hospital at the Department of Obstetrics and Gynecology. In the county of Uppsala, approximately 4000 infants are born annually. The study protocol was approved by the Regional Research and Ethics Committee of Uppsala.

### Study population and data collection

All eligible women giving birth at Uppsala University Hospital between May 2006 and June 2007 were asked within the first hours after birth if they wanted to participate in the UPPSAT study. The women received both written and oral information about the study and were given a first questionnaire to fill out five days postpartum. The questionnaire was returned to the hospital by mail or, if the woman was still in the hospital, filled in and handed to their midwife. A second questionnaire was sent to the women by mail six weeks postpartum. A third questionnaire was sent out six months postpartum. As an addition to the third questionnaire, a number of women received an extensive breastfeeding questionnaire at six months postpartum.

Exclusion criteria for the UPPSAT study were not being able to speak, write or read in Swedish, protected identity, stillbirths and if the baby was admitted to the neonatal care unit immediately after birth (441 women excluded). Two thousand four hundred and ninety-three women gave written consent to participate. The additional breastfeeding questionnaire was sent out six months postpartum to 1569 women and 879 women returned it. For the current study, women giving birth prematurely (before 37 weeks of gestation), women not initiating breastfeeding at all and women giving birth to twins were excluded (86 women excluded). Women not completing questions on breastfeeding duration were also excluded (114 women excluded), which gave a total number of 679 participating women.

### Outcome measures

Mothers who had given their newborns anything else than breast milk at any point from birth to two months postpartum, as well as women ceasing breastfeeding within in this time, were classified in the “EBF < two months”-group. The two-month time point was chosen in order not to interfere with guidelines on supplemental foods. Although Swedish women, in general, are recommended to breastfeed exclusively for six months, the Swedish National Food Agency states that parents can start offering their babies small portions of solid food from four months age [3]. The Swedish National Board of Health and Welfare prepares statistics of breastfeeding in Sweden and shows figures of breastfeeding for two, four and six months [15] which was also the reason for choosing two months as the time point for this study.

### Study variables

Questions about various background and antenatal factors (age, body mass index (BMI) before pregnancy, family status (married/cohabitant vs single), educational level (university/college vs high school), smoking during pregnancy, previous psychiatric or psychological contact, emotional distress during pregnancy and experience of giving birth) were included in the first questionnaire. The question about subjective emotional wellbeing during pregnancy “*How did you feel emotionally during pregnancy*?” [17] was dichotomized as non-distressed (more happy than usual, as happy as usual) or distressed (somewhat low mood, low mood). So was the variable “experience of giving birth,” which was dichotomized as positive (excellent, good, okay) or negative (bad or awful).

A second questionnaire was sent to the women six weeks after birth, containing *inter alia* the Stressful Life Event (SLE) scale by Rosengren et al. [18] The SLE variable was dichotomized into none or one SLE versus two or more SLEs during the past year, since one SLE could be considered normal life stress.

The additional breastfeeding questionnaire, sent as a complement to the third questionnaire and answered by the women six months postpartum, included a series of questions on initiation of formula supplementation and solid food as *“At what age did your baby receive formula for the first time*?*”* and *“If you don’t breastfeed now*, *at what age (of the baby) did you stop*?*”* It also included statements such as *“The first breastfeeding session was a positive experience to me”* with yes/no alternative answers. Information on the hands-on approach during the first breastfeeding session was also included, as was the place for the first breastfeeding session (delivery ward versus the maternity ward, in which the latter consequently indicates a postponed first breastfeeding). Data on gestational age, mode of giving birth and the use of anesthetics during childbirth were retrieved from the medical records.

### Statistical analyses

SPSS version 20.0 and R version 3.1 were used for the statistical analyses. The level of statistical significance was set at a p-value of 0.05.

Univariable analyses were performed to assess the possible predictors of EBF for less than two months postpartum in contrast to EBF lasting more than two months. Possible predictors were socio-demographic and obstetric factors, caregiving routines and the mothers’ subjective experiences of childbirth and the first breastfeeding session. Odds ratios (OR) and 95% confidence intervals (CI) were calculated using the Mantel-Haenszel procedure. A multivariable logistic regression model was fitted to estimate the specific effect of the background variables on EBF < two months postpartum. The variables were included based on review of the previous literature and for their clinical relevance. Variables included in the model were BMI, parity, subjective emotional distress during pregnancy, giving birth by cesarean section, use of epidural anesthesia, the hands-on approach during the first breastfeeding session and postponed first breastfeeding session. Adjusted OR and 95% CI were estimated. The C index, equivalent to the area under the receiver operative characteristics (ROC) curve, was used to assess the discriminative ability of the model. Based on the results of the multivariable logistic regression, a nomogram was created by rescaling the regression coefficients to a scale from one to ten. That is, the nomogram is a re-representation of the logistic regression model, in order to make it easier to apply the model without having to use the actual regression equation. Internal validation of the final model, both regarding calibration and discrimination, was done using bootstrap methods[19]. In each bootstrap sample the model was refitted and then evaluated on the full dataset. The apparent performance, defined as the performance of the final model evaluated on the full data, was compared with the test performance, defined as the average performance of the models in the bootstrap samples evaluated on the full data. The optimism of the initial model was defined as the difference between the apparent and the test performance.

## Results

### Description of sample

Among the participating women (n = 679), the mean age was 30.7 years (standard deviation [SD] 4.3 years). Twenty-eight percent had a BMI equal to or over 25 kg/m^2^ and 99% of the women were married or cohabitant. Sixty-two percent of the women had a college or university education. Forty-six percent were primiparous. Twenty-three percent of the mothers reported subjective emotional distress during pregnancy and twenty-three percent had a previous contact with psychiatric or psychological healthcare services.

Thirteen percent of the women gave birth by cesarean section. Thirty-six percent of the women reported use of the hands-on approach during their first breastfeeding session. Ninety-four percent reported a positive experience of breastfeeding for the first time. Seventy-nine percent of the women reported that their first breastfeeding session took place in the delivery ward, whereas the others reported postponed breastfeeding, taking place in the maternity ward. Seventy-seven percent of the women breastfed exclusively for at least two months.

### Univariable analysis

The univariable analyses are displayed in Table 1. The univariable results indicate an increased risk for EBF lasting < two months postpartum for women with a high BMI, first time mothers, those with a previous contact with psychiatric or psychological healthcare services and those who experienced subjective emotional distress during pregnancy.

**Table 1:** Sociodemographic characteristics and obstetric variables in association with exclusive breastfeeding lasting less than two months postpartum.

Variable		Total n(%)	EBF ≥two months n(%)	EBF <two months n(%)	OR for EBF < two months	95% CI
Sociodemographic characteristics						
Age (yr)	<25	32 (6.6)	28 (87.5)	4 (12.5)	0.45	0.15–1.32
	25–34	369 (75.9)	280 (75.9)	89 (24.1)	1.0	1
	≥35	85 (17.5)	68 (80.0)	17 (20.0)	0.79	0.44–1.41
BMI^a^ (kg/m^2^)	<25	376 (72.2)	296 (78.7)	80 (21.3)	1.0	1
	≥25	145 (29.8)	100 (69.0)	45 (31.0)	1.67	1.08–2.56
Married/cohabitant	Yes	574 (99.1)	441 (76.8)	133 (23.2)	1.0	1
	No	5 (0.9)	5 (100)	0 (0)	-	-
Education level	High	361 (62.0)	282 (78.1)	79 (21.9)	1.0	1
	Low	217 (38.0)	163 (75.1)	54 (24.9)	1.18	0.80–1.76
Parity	Multi	364 (53.7)	309 (84.9)	55 (15.1)	1.0	1
	Primi	314 (46.3)	213 (67.8)	101 (32.2)	2.66	1.84–3.86
Smoking during pregnancy	No	513 (97.3)	405 (78.9)	108 (21.1)	1.0	1
	Yes	14 (2.7)	9 (64.3)	5 (35.7)	2.08	0.68–6.35
Stressful life event^b^	No	430 (78.2)	336 (78.1)	94 (21.9)	1.0	1
	Yes	120 (21.8)	89 (74.2)	31 (25.8)	1.25	0.78–1.99
Previous psychiatric contact	No	449 (77.3)	357 (79.5)	92 (20.5)	1.0	1
	Yes	132 (22.7)	90 (68.2)	42 (31.8)	1.81	1.18–2.79
Emotional distress during pregnancy	No	439 (77.4)	351 (80.0)	88 (20.0)	1.0	1
	Yes	128 (22.6)	87 (68.0)	41 (32.0)	1.88	1.21–2.92
Obstetric variables						
Cesarean section	No	593 (87.3)	473 (79.8)	120 (20.2)	1.0	1
	Yes	86 (12.7)	50 (58.1)	36 (41.9)	2.84	1.77–4.55
Elective Cesarean section	No	593 (94.4)	473 (79.8)	120 (20.2)	1.0	1
	Yes	35 (5.6)	22 (62.9)	13 (37.1)	2.33	1.14–4.76
Emergency cesarean section	No	593 (92.1)	473 (79.8)	120 (20.2)	1.0	1
	Yes	51 (7.9)	28 (54.9)	23 (45.1)	3.24	1.80–5.82
EDA^c^ during delivery	No	453 (66.7)	366 (80.8)	87 (19.2)	1.0	1
	Yes	226 (33.3)	157 (69.5)	69 (30.5)	1.85	1.28–2.67
Entonox during delivery	No	165 (24.3)	127 (77.0)	38 (23.0)	1.0	1
	Yes	514 (75.7)	396 (77.0)	118 (23.0)	0.99	0.66–1.51
Caregiving routines						
Hands-on approach^d^	No	429 (64.1)	354 (82.5)	75 (17.5)	1.0	1
	Yes	240 (35.9)	161 (67.1)	79 (32.9)	2.32	1.61–3.34
Place/setting during first breastfeeding	Delivery ward	537 (79.1)	439 (81.8)	98 (18.2)	1.0	1
	Maternity ward^e^	142 (20.9)	84 (59.2)	58 (40.8)	3.09	2.07–4.61
Experiences						
Positive experience of the first breastfeeding	Yes	627 (93.9)	497 (79.3)	130 (20.7)	1.0	1
	No	41 (6.1)	19 (46.3)	22 (53.7)	4.43	2.33–8.42
Positive experience of giving birth	Yes	439 (89.0)	348 (79.3)	91 (20.7)	1.0	1
	No	54 (11.0)	36 (66.7)	18 (33.3)	1.91	1.04–3.52

^a^ Body Mass Index

^b^ 6 weeks postpartum

^c^ Epidural local anaesthetics

^d^ During first breastfeeding session

^e^ Maternity ward, which consequently indicates postponed first breastfeeding session.

Women who gave birth by cesarean section and/or used epidural anesthesia (EDA) during labor also had a higher risk for EBF < two months postpartum. Moreover, women receiving the hands-on approach and women reporting that their first breastfeeding session took place in the maternity ward (postponed breastfeeding) were at greater risk to report EBF < two months postpartum. If the women reported a more negative experience of the first breastfeeding session and/or a more negative experience of giving birth they were less likely to be breastfeeding exclusively at two months postpartum.

### Multivariable analysis

A multivariable logistic regression model was fitted to estimate the specific effect of the background variables on EBF < two months postpartum. Variables included in the model were BMI, parity, subjective emotional distress during pregnancy, giving birth by cesarean section, epidural anesthesia (EDA), the hands-on approach during the first breastfeeding session and postponed first breastfeeding session. Variables were included for their clinical relevance and/or based on review on the previous literature. In the multivariable logistic regression analysis, displayed in Table 2, primiparity, reporting subjective emotional distress during pregnancy and giving birth by cesarean section were all independently associated with EBF < two months postpartum. The association between BMI, use of EDA during birth, the hands-on approach during the first breastfeeding session and postponed breastfeeding initiation did not reach statistical significance. Stratification of the data for mode of delivery did not influence the results. The multivariable model had a C index of 0.72. The internal bootstrap validation of the model resulted in an optimism-adjusted C index of 0.70 and a calibration slope of 0.91 indicating that the model is likely to perform well for predictions in similar settings.

**Table 2:** Multivariable logistic regression model for factors associated with EBF lasting less than two months postpartum.

Variable		Adjusted OR for EBF <two months (95% CI)
BMI^a^ (kg/m^2^)	<25	1
	≥25	1.45 (0.90–2.32)
Parity	Multipara	1
	Primipara	2.15 (1.32–3.49)
Emotional distress during pregnancy	No	1
	Yes	2.21 (1.35–3.62)
Cesarean section	No	1
	Yes	2.63 (1.34–5.17)
EDA^b^ during pregnancy	No	1
	Yes	1.55 (0.98–2.46)
Hands-on approach^c^	No	1
	Yes	1.34 (0.83–2.16)
Place/setting during first breastfeeding	Delivery ward	1
	Maternity ward^d^	1.75 (0.99–3.09)

^a^ Body Mass Index

^b^Epidural local anaesthetics

^c^During first breastfeeding session

^d^Maternity ward, which consequently indicates postponed first breastfeeding session.

**Fig 1:**
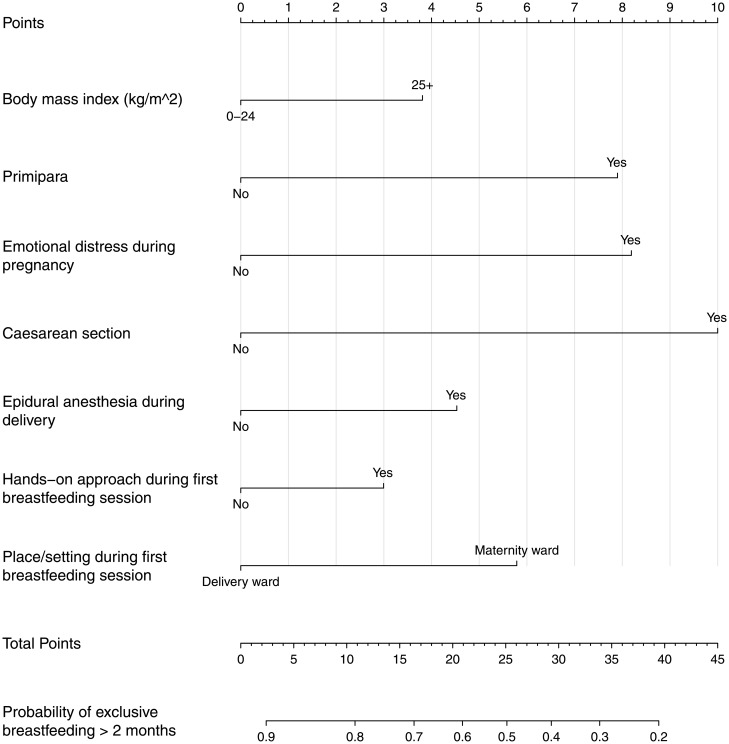
Nomogram for probability of exclusive breastfeeding > two months postpartum.

Fig 1 shows a nomogram, with variables from the multivariable analysis, weighted in order to reflect their effect size in predicting EBF equal to or more than two months postpartum. That is, using the nomogram is equivalent to estimating the probability of EBF using the logistic regression equation. For each predictor, a point is assigned on the 0–10 scale at the top. The sum of points gives a total score, which reflects the probability of EBF at two months postpartum. As an example, consider a first time mother (8 points) reporting emotional distress during pregnancy (8 points) who gave birth by cesarean section (10 points), and had a postponed breastfeeding initiation (6 points). The total number of points is 32, corresponding to a probability of EBF ≥2 months of approximately 37%. In the case of missing information on one of the variables, e.g. emotional distress during pregnancy (ED), two predictions could be made, one for ED=“No” and one for ED=“Yes”.

Explanatory matter: Consider a first time mother (8 points) reporting emotional distress during pregnancy (8 points) who gave birth by cesarean section (10 points). The total number of points is 26, corresponding to a probability of exclusive breastfeeding ≥ two months of approximately 50%.

## Discussion

The main findings of this population-based longitudinal study on EBF duration was that factors independently associated with EBF < two months postpartum were being a first time mother, subjective emotional distress during pregnancy and giving birth by cesarean section. These results are in concordance with previous studies. However, this is, to our knowledge, the first study using a nomogram to provide a tool for healthcare professionals working in postnatal care.

There are previous researchers who aimed to identify women at risk for an early cessation of EBF. In a recent article, conducted in Italy by Lindau et al [11], women giving birth by a planned cesarean section and women with psychological stress conditions were at greater risk for shorter EBF duration, in the multivariate analyses. Compared to our study, this Italian study had fewer participants (542) and did not take into consideration whether the woman was a first time mother. Breastfeeding duration has previously been shown to be shorter among first time mothers [20], which was confirmed in our data. It has been noted that first time mothers have a high use of EDA [14], and studies show that EDA can affect the infant in a way that postpones the first breastfeeding session [21,22]. The borderline association in the multivariate analysis between a higher maternal BMI and EBF < two months postpartum is in line with previous studies [9,10]. Subjective emotional distress was independently associated with EBF < two months postpartum. Emotional distress during pregnancy might be problematic to identify, because pregnant women seldom disclose emotional distress [23]. In antenatal care in Sweden, there is no national consensus on how to screen for depression and anxiety. However, depending on local guidelines, midwives and obstetricians address this issue and document it. The association between subjective emotional distress during pregnancy and EBF < two months postpartum may be due to low self-esteem, as well as a result of depression and/or anxiety prior to or during pregnancy and postpartum, factors known to negatively affect breastfeeding duration [11–13].

Compared to vaginal birth, cesarean section includes several aspects that can affect breastfeeding rates, such as anesthesia [10], and separation [24], between mother and newborn. In Uppsala University Hospital, the newborn is taken out of the operating theatre as a routine, for observation and assessment, and when the mother is under observation postoperatively, the separation is extended, and the newborn will be cared for by the mother’s partner in the maternity ward. This separation leads to disturbance of the important early skin-to-skin contact between infant and mother, thus negatively affecting the possibility for a successful first breastfeeding [25]. Infants born through cesarean section are more often distressed and in need of immediate assistance during the first minutes of life [26]. Cesarean section is often associated with more pain in the first days postpartum, which can affect the mother’s well-being and breastfeeding [27].

It is well known that initiation of breastfeeding within the first hour of the newborn’s life—which is recommended by the WHO—is an important factor for successful breastfeeding in the long run [5–7]. In Uppsala University Hospital, where the current study took place, a new mother was recommended to stay in the maternity ward for further care and support if there were one or more obstetric complications and/or if a successful first breastfeeding session was not observed in the delivery ward within two hours after birth. The borderline association between postponed breastfeeding initiation and EBF < two months postpartum is important, since the time between giving birth and initiation of breastfeeding is a routine possible to modify, if prioritized. Even if a woman gives birth by cesarean section, or has experienced other invasive procedures after birth, it should be possible to avoid separation or at least shorten the time of separation, via new caregiving routines. During recent years, the obstetric operation ward at Uppsala University Hospital has taken steps in this direction and improved care giving after cesareans by allowing the baby to stay with the mother in the recovery ward after the operation, thus improving early skin-to-skin contact, and hopefully enhancing the chance for an early first breastfeeding session.

Another problem with postponed breastfeeding initiation is that it may result in the use of the hands-on approach. A postponed first breastfeeding session will, most likely, render the health care professionals more prone to use the hands-on approach, which might influence the woman negatively, even in a longer perspective. In a previous study, the results showed that women exposed to the hands-on approach reported the first breastfeeding session as a more negative experience and that the hands-on approach was more common if the woman did not have the opportunity to breastfeed while in the delivery ward [16]. The hands-on approach was, in the current study, statistically significant for EBF < two months in the univariable analyses, though not statistically significant in the multivariable model. Nevertheless, the use of the hands-on approach should be questioned.

### Strengths and limitations

The number of participating women, as well as the number of studied variables on an individual level, which gives the possibility for adjusted analyses, is among the strengths of this study. It can be argued that the retrospective design of this study, leaving the women to answer questions about events of the first hours postpartum and breastfeeding duration six months postpartum, poses an eventual problem of recall bias. Nevertheless, it has been shown that women successfully recall what happened during the process of giving birth and the following hours, even a long time later [28]. Agampodi and co-authors [29] argued that retrospective evaluation methods systematically overestimate the duration of breastfeeding, but our prevalence of 77% women breastfeeding exclusively for at least two months is similar to that of breastfeeding rates in Uppsala county during the same year (76,2%) [30]. A possible limitation of this study is that a question about intention to breastfeed was not directly posed, as we assumed that nearly all mothers had planned to breastfeed. The question on emotional distress during pregnancy is not validated, although it is the question recommended for midwives in antenatal care to ask during pregnancy, according to the Swedish Society of Obstetrics and Gynecology [17]. Another limitation might refer to the fact that postponed breastfeeding was defined by which place the first breastfeeding session occurred. Due to the hospital routines, we know that women who answered that their first breastfeeding session took place in the maternity ward would have had a postponed breastfeeding initiation of at least two hours. One could also speculate if it would be of interest to separate emergency cesarean section from planned cesarean section. This was indeed performed in the univariable analyses, and showed that both these groups had statistically significantly higher odds for EBF < two months. Therefore, these groups were analyzed together in the multivariable analyses.

The use of a nomogram is, to our knowledge, a new approach in this research area, identifying women who need targeted breastfeeding support. Using a nomogram as a graphical display of the multivariate logistic regression results might be useful for risk assessment and to prioritize targeted breastfeeding support. For example, a woman reporting emotional distress during pregnancy, giving birth to her first child via cesarean section and having a postponed first breastfeeding session would score 32 points on the scale, corresponding to a probability of only 37% of breastfeeding exclusively longer than two months. The monogram might give valuable information for the healthcare professional on how to prioritize, when trying to identify women in need of additional breastfeeding support.

## Conclusions

Primiparity, emotional distress during pregnancy, and giving birth by cesarean section were identified as independent factors for EBF < two months postpartum. To promote successful breastfeeding, adequate breastfeeding support after identification of risk factors should be initiated during pregnancy, intensified right after delivery and followed up postpartum. As a clinical or educational tool, we introduce a nomogram, which can help caregivers pay attention and target women within certain risk groups. These risk factors could be used in future intervention studies, targeting women at high risk for shorter exclusive breastfeeding duration. Further research is also needed, in order to replicate this study in other settings.

## Supporting information

S1 File Questionnaires. Questionnaires on sociodemographic characteristics, stressful life events and breastfeeding.
(DOCX)

S2 File Data set. (SAV)
