# Supplementary material for: Risk factors for exclusive breastfeeding lasting less than two months—Identifying women in need of targeted breastfeeding support
Source: PLoS One. 2017 Jun 14;12(6):e0179402. doi: 10.1371/journal.pone.0179402 (PMC5470694; doi:10.1371/journal.pone.0179402)
Supplement: S1 File — Questionnaires on sociodemographic characteristics, stressful life events and breastfeeding. (DOCX) [file pone.0179402.s001.docx]

**Questionnaire (S:Swedish, E:English)**

**Questionnaire 1, to be filled out five days postpartum:**

S: Vilken är din födelsedag   ÅR: MÅN: DATUM:

E: Please state your date of birth YEAR: MONTH: DATE:

S: Är du: Gift Sammanboende Änka Skild

E: Are you: Married Cohabitant Widowed Divorced

S: Vilken är din högsta utbildning?

Folkskola Grundskola Gymnasium Folkhögskola Universitet

E: Please state your highest education:

Primary school - Junior High school - High school - College - University

S: Vad vägde du innan du blev gravid (kg)?

E: Please state your pre-pregnancy weight (in kilograms)

S: Har du någonsin varit hos en

Psykiatriker - psykolog -Auroramottagningen?

E: Have you ever consulted a psychiatrist, psychologist or a midwife specially trained in dealing with severe fear of childbirth?

S: Hur mådde du under graviditeten? Gladare än vanligt, precis som vanligt, lite nedstämd, nedstämd

E: How did you feel emotionally during pregnancy? More happy than usual, happy as usual, somewhat low mood, low mood

S: Hur skulle du beskriva förlossningsupplevelsen? Underbart bra OK dålig hemsk

E: Please describe how you experienced giving birth: Excellent good ok bad awful

**Questionnaire 2 (The Stressful Life Event scale), to be filled out at six weeks postpartum, by ticking a box after each statement:**

S: Under det senaste året, har det hänt något av följande:

E: During the past year, has something like this happened:

S: Allvarlig sjukdom hos familjemedlem?

E: Serious illness in family member?

S: Stark oro för en familjemedlem?

E: Strong concern for a family member?

S: Död i familjen?

E: Death of a family member?

S: Separation eller skilsmässa?

E: Separation or divorce?

S: Har du blivit tvungen att byta bostad?

E: Forced to move from house?

S: Har du blivit tvungen att byta jobb?

E: Forced to change job?

S: Har du känt dig överflödig?

E: Been made redundant?

S: Har du känt dig osäker på jobbet?

E: Feelings of insecurity at work?

S: Allvarliga ekonomiska besvär?

E: Serious financial trouble?

S: Problem med lagen?

E: Been legally prosecuted?

**The additional breastfeeding questionnaire, to be filled in six months postpartum:**

S: Första gången jag ammade mitt barn var på förlossningsavdelningen, BB, neonatalavdelningen, annat

E: I breastfed for the first time in the delivery ward, the maternity ward, the NICU, elsewhere

S: Första gången jag ammade hjälpte vårdpersonalen mig med amningen med sina händer och kopplade ihop mitt bröst med barnets mun Ja Nej

E: At the first breastfeeding session the healthcare professionals helped me breastfeed by using their hands to attach my breast with the baby’s mouth Yes No

S: Jag upplevde första amningstillfället positivt Ja Nej

E: The first breastfeeding session was a positive experience to me Yes No

S: Vid vilken ålder fick ditt barn ersättning för första gången?

E: At what age did your baby receive formula for the first time?

S: Om du inte ammar nu, när slutade du amma?

E: If you don’t breastfeed now, at what age (of the baby) did you stop?

S: Barnet fick tillägg på BB Ja Nej

E: Did your baby receive formula in the maternity ward?
